# Supplementary material for: In-Silico Characterization of Estrogen Reactivating β-Glucuronidase Enzyme in GIT Associated Microbiota of Normal Human and Breast Cancer Patients
Source: Genes (Basel). 2022 Aug 27;13(9):1545. doi: 10.3390/genes13091545 (PMC9498756; doi:10.3390/genes13091545)
Supplement: Supplementary file 1 [file genes-13-01545-s001.zip › genes-1882403-supplementary.pdf]

Supplementary Data Table S1: Names, UniProt Accession IDs and sequences of probiotics documented in present study

Bacterial name	UniProt Accession ID	Sequence of uidA protein
Lactobacillus rhamnosus	A0A7Y7QF62	METSLLYPVTNDQRTDQKLDGLWQFKFDEAGEGEKSGWETGFHDG<br>VSMPVPASFNDFFTDKASREYTGDFWYSRNFFVPSAAKGKALFLRFD<br>AVTHRATIFVNGKEIRTHEGGFLPFAADISEAVKYGAENTVVVKGNN<br>ELSREALPAGDTITLRNGKKMVRPFFDFYNYSGLNRSVHLLSLPQER<br>VLDYTTTFALAGNDATVNYTVETNGDAPVTVSLADADGQVVATAQ<br>GKQGALQVQNAHLWQVRNAYLYTLTIQLGDDTQTPLDTYTDRIGIR<br>TIKISGTDILVNDKPIYLKGFGRHEDSPFAGRAFDLNVEKKDFALMK<br>WIGANSFRTSHYPYDEQVYKIADEEGFLLTDEVPAVGFKMAAAAFL<br>GGLNQSFFKGPWLKKLHERHIDQIRDLIKRDKNHPSVLAWSLFNEPD<br>TIDENAVPYFKQIFDESKDLDPQGRPRTFTLSEDDTIETSKVLDFPDFY<br>MLNRYPGWYHFGGYQISDGEAGLRDEMDKWQKAGVKKPVVFTEF<br>GADTEAGLHKLPSVMWTEEYQVEVLKMFSRVFDDYDFIKGEQVWN<br>LADFQTVEGNMRVNGNKKGIFTRDRQPKAAAFFYHDRWNKLPLDY<br>KAK
Roseburia intestinalis	A0A6L6KZP5	MLYPQENKIRGIIDLNGVWDFALGTTEEQGDAVTLPEQMESIAVPAS<br>YNDQKDDIAYRNHYGYAYYRRNITVPSYYKGQRLVLRFDAVTHFA<br>KIYLNGVLLTQHKGGFLPFEVDITDKLCAGESAELVVAVDNRINHST<br>LPVGNEEGTSFMGADNAGVPGVEAAKRWRKPQNLPNFDFFNYAGIN<br>RPVRIYTTPKAYIKDVTLVTDIRGTDGIVNYQVKTSDTDGQEVVLQIL<br>DANGNEVAQAKGTSGEIVIPDAKLWEPYPGTPYLYTAAVTFGDDYY<br>EEPFGVRTVRVEGTSFLINGKPFYFKGFGKHEDSAFHGRGMDVCLDV<br>KDVNLIHWLHANSFRTSHYPYAEEMYRLCDREGIVIIDEVPAVGIGA<br>GAGINPYETFPIREHHEQVIKDMIARDKNHPSVVMWSLGNEPDTENF<br>PESAYEYWHSLYELAHETDPSDRPVTLVCCQNNYEKDIVTRSMDVV<br>CINRYYGWYNLSGDLDAACYGLNLELDFWESQNKPVMITEYGADA<br>VAGIHECVPEMFSEEFQVEFYKRQNAQFDKRKFFIGEHVWNFADFAT<br>VQGCMRVDGNKKGLFTRERRPKMAAHYFKERWGEIPNFEYKG
		MQKTDFNRDWTVQKDGSNEILHVNLPDDAMIREERSKENKTASASA<br>YFAGGKYIYTKIFDLSENETRQTLILEFEAVYQNATVFLNGRQVAEHP

Coprococcus comes	A0A173S7E7	YGYTNFFVDITGKVIAGTNELKVVADNADVPNSRWYSGSGIYREVHLYRSGSSYIRPEGLKVQIVDLNTIHIFTDAVMQPDEKIVLEIFNDTGKIVSSEGTDVTITIPDAHLWSAEDPYLYTCKATLLQNGTAIDTARTSFGIRTLSWGKDGFLVNSKSVLFRGACIHHDNGVLGACGFHDAECRRVRILKEAGFNAIRSSHNPISKAMLDACDQLGMYIIDEAFDMWLIKKNPYDYAGETFSKWWKADIAAMISKDYNHSSVVMYSVGNEITELGLADGQEQARIMTEFCHTKDHTRPVTAGINLMLATMAGSKKSIYGTDEDGKVKDSGSGGLDNAPTSEFFNIMMNKMGGLINKAAKTKKATAIAEIMSGIFDIPGYNYASSRYKIDARNHPEQATTGSETLPQTLYDNWQLVKSIPTMTGDFMWTGYDYLGESGIGTIQYKDKKTKQNADPGLIISGGAGIIDICGKKRPEVGWSKIIWGLQKTPTIGVDPYTRTDYFQSLSMWRVTDAVESWSWEGCEGKKASVTIYSDADKIELLVNGKSAGKKKPKKDIAKFRKIPYEAGKIEAIAYDSSGKETARTTLVSATGKTSIKLTPETTNLCANGQDLCFLNIDLIGENGITKSSVDQELKIEISGPATLQGYGSARPNVEESFCNDTFKTFYGKSLAVIRAGYEPGTVTVKVSGKGLDTQELTLNIC
Faecalibacterium prausnitzii 1	A0A6A8KDD0	MLYPEQNEARLKLSLDGTWAFALGSCVEDQFDPAKPLPDAQPIAVPASYNDQNDQTTALRRHYGWAWYQRKVTLPTFCAGQRVVLRFGSVTHTAKVWLNGKLIAQHKGGFTPFEADVTALLRPGETVLLTVACDNRVNHSTLPVGNEDGQLAFFGSDNAGIPSVEAAKRTAAPQNRPNFDFFNYAGIHRPVWLYTTPKEYIEDVTVVPAVDGTVQYAVKTTGSAPVHVMVLDADGNAVASAEGVEGTLTIPEVHLWEPRPGTPYLYTLHITCGADVYDQSFGVRSIEVRGTQVLLNGKPLYFKGFCKHEDFTAHGRGFDPVLNVKDVNLIHWANANAVRTSHYPYAEEFYDLCDREGILVMDETPAVGIGGGAAVNPYKEYPLAEHHRQVLAEMIHRDKNHPCVVLWSLGNEPDLEHFPQDAYDYWHPLYELAHQLDPQNRPVTLVCCQNDYTKDITTRTMDIVCINRYYGWYNLSGDMDAACYGLNQELDFWAEQHKPVMMSEYGADTVAGLHTAGAEMFSEEFQVEFYRRLDAEFDKRPWFVGEFVWNFADYDTVQGPMRVDGNKKGLFTRDRRPKLGMHFLRQRWAEIPTFGFKE
		MNRSLLYPRATTTRRLIGLDGMWRFSFDPESKGVEAGWALELPSSLSMPVPASFCDLFTDRASREYCGDFWYETSFFVPAEWSGWDIVLRFGSVTHRARVFVNGVEVAQHEGGFLPFDATVTNIVRYNQFNKLSVLANNELSETMLPAGTTRTLADGRKIAAPYFDFYNYAGIHRPVWLMALPKE

Faecalibacterium prausnitzii 2	A0A844DSU7	RVLDYSTRYRLTETGAEIDYTVSTNGPHPVTVELYDGTTRVAESSGTTGTLVVKNAKLWNVHAAYLYDLVIRIHEGSAVVDEYLDRIGIRTFEIRHGRFLLNGSPVYLRGFGRHEDADIRGRGLDLPTVKRDFELMKWIGANCFRTSHYPYAEEIYQMADEEGFLIIDEVPAVGFMQSTANFLAANQGNGRQQGFFEKETTPALLKNHKAALSDMIDRDKNHPSVIAWSLLNEPQCTSAGTEEYFKPLFELARRLDPQKRPRTYTVLMTSLPDTSKGQRFADFVSLNRYYGWYVLGGAGLADAEAAFHHEMDGWAKVLHGRPLIFTEYGTDNLSGAHKLPSVMWSAEYQNEYLEMTHAVFDHYDFVQGELVWNFADFQTTEGILRVDGNKKGIFTRQRQPKDAAYLFRERWTTLPVDFKKRKK
Faecalibacterium prausnitzii 3	A0A173S5S4	MRSIAKLMQDWQFTGPDGKTTLVELPHTWNAKDGQDGGNDYWRGTCTYSTTFAAPAFDAASQEVWLQFEGVNSSAKVLLNGRNICTHDGGYSTFRVHISDLLAKDNQLTVEVDNSINDRVYPQKADFTFYGGIYRDISLMVVSRNHIALGHFGDTGVKITPALKDGRADIRVETLVEGEGAVSVELQDAAGSIVARAEGADAQLHLAAPHLWDGVKDPYLYTCVVRLSSDGTVVDEVSTRVGLRTFSVDSRNGFFLNGRPYPLHGVSRHQDRKGVGNAITREMHDEDMALIRELGANTVRLAHYQHDQYFYDLCDQYGMVVWAEIPYISEHLPNGRANTVSQMKELIYQNYNHPCIVCWGVSNEITISTRDKADMLDNHRELNDLCHKMDSTRLTTLACYAMCGPFNPVAHITDLVSWNLYLGWYVPGLFLNDLWMDFFHLVYPGRPLGFSEYGAEGMPNLHSSKPRRGDHTEEYQAKYHEYMLRCFDRHKWMWATHVWNMFDFAADARDQGGEPGMNHKGLVTFDRKTRKDSFYLYKAWWSDENFVHICSKRFTDRTEREMEVKVYSNQKSVALYVNGEKAGEQTGEHVFSFRVPLTGEIEVKAVAGDCTDTASFRHVDTPNPSYKLVKTKSKSANWV
Propionibacterium acnes	A0A533IM53	MMLRPQDTATRDTKCLSGMWDFAFDPEDRGQTERWFTQLLPERTEMAVPASYQDLSTDPATRDYVGPVWYQREVRIPRGWVGNRVVVHFESATHAATVWINDIEVVSHVGGYLPFEVDITDHVRAGKKCRLTVRVDNRLSFQTIPPGIIVDSPEGPKQKYWHDFFNYAGIHRDVWLCSRPQVHVEDVTITTDIDGGDGIVTWSVLSHNADLLATRVTIFDDEGNVVAEETGANAQTRIPSVHLWQPGQGYLYEAEISLVNGDEVIDAYRQAFGVRTIRVDGTRLLINGEPVYLTGFGMHEDHQTIGKAHNDALMLRDAACLEWVGANSLRTSHYPYSEHILDYADRHGLLVIDETPAVGMNMGLGGGI

		FGTQGYTTFSAETINDETQKVHAQVIRDLIDRDKNHPSIIIWSIANEPE<br>SETEAAENYFRPLFDVAHEADPSRPVSFVNVMLAPFGKCRVSQYSDI<br>LLLNRYYGWYVDTGDLAAAERHWREEMAGWASENKPIIITEYGAD<br>TMPGLHQIPAEPWSEEYQVEVLKMNERIFDSFDAVIGEQIWNFADFA<br>TTSGTMRVGGNRKGIFTRDRQPKSAAFHLRKRWRGVKQ
Methylococcaceae bacterium	A0A7Y4RRN1	MLYPQQNISRNKFDLSGIWDFKIDPDRMGERNGWHTGLDGARPIAV<br>PGSWNEQYEDIYNYLGLAWYVKSTYVPQYWQGQRVFLRVGSACYF<br>STIYVNGIKMGSHEGGHLPFVFEVTDHLCWKTENTIAISVENDLMPT<br>RVPSGNMSSPLMPQSCFPRTTYDFFPFAGIHRPVVLYSVPPVYIEDITV<br>ITGINGLDGTVKVRVQLNSADTIQGSVHIKGDEVTFTAKLMFKDGLA<br>EAHLIVHNANFWSDKTPYLYDLTVQTEQDQYTLKVGVRTVKVQGG<br>QIFLNDKPIHLNGYGRHEDFYASGKGLNQPLMVKDYQLMRWTGAN<br>SYRTSHYPYSEEEMQLADREGFLIINEIPAVSLQFDNDENIAIRLRTCL<br>QQVDELITRDKNHASVVMWCIANEPLIAQPGLGVSVSSNVFSVAKG<br>KEFLGTLMQRVRTLDPTRLVTFVALMGSPSSWVEQCDVICMNRYW<br>GWYILGGELNKALISLEQELDSAWETWHKPIMITEFGADTMAGMHG<br>HPNAMWTEEYQAEFVRGYLTVAARKDFIAGMQVWNFADFASIQSI<br>MRVGGMNMKGIFTRGRTPKMAAHVLREFWIK
Staphylococcus xylosus	A0A8B5JKU8	MLYPVVNEFRSIIDLNGIWRFKLEGNEDQIDVSQPLNTEQVMAVPGS<br>FNDQGVTANIRNHVGNVWYERTFTVPNVLNNERIVLRFGSATHKAT<br>VYIDGNEVTSNQGGFLPFEVTLEKAYTVGTHRLTVCVNNILDETTLP<br>VGDYSETTDAQGNMIKKNIPNFDFFNYAGLHRPVKIYTTPKTHIQNIE<br>IVPEVQGDDAFVNYKVSTNHSRGAVKVQLVDEDKQVIAESTGVEGT<br>IKVQNPHLWQPLKAYLYHLEVSLIEDEEIIDTYAERFGIRSVKVDKGK<br>FLINGEPFYFKGFGKHEDSYYHGRGMDEVVNVLDFNLMKWIGANSF<br>RTSHYPYSEEMMRLADEQGIVVIDETTAVGVHLNFNAILTGEKERNT<br>FKEIGTKAAHEAVIKGLIERDKNHACVVMWSIANEPASDEQGAKAY<br>FEPLVNLARTCDPQQRPVTIVTILTSQPDTCQVQELVDVLCLNRYYG<br>WYTQTADLKAAKLALAEELDGWSIKQPDKPIMFTEYGADTVAGMH<br>ALNDELFTEEYQIRYYEANHEVMDKYPQFIGEQVWNFADFETSSGII<br>RVQGNKKGIFTRERRPKAVAHYFKERWSNIPDFGYKI

Streptococcus suis	G7SFL4	MLYPIQTKTRSVYSLNGIWKFKQGNNDVHTLLDTDEVMVVPSSFND<br>LVVDKVKRRFVGDNWYELMVALPVVSADEELVVRFGSVTHQAKV<br>YADGQLIGEHKGGFTPFECLIPSNLYDADQFRLTVCANNELNYTTLP<br>VGNYSEEVDENGQIVKTVKENFDFFNYAGIQRTVHLYKRPKNRIEDI<br>VIRTELNQDLTQAVVNVDVKTVGQYDSIRMSILDQEGQEVGLLENG<br>QMVIEHPRLWEVLDAYLYTAKVELVDGENLLDTYSEQFGIRSVAVE<br>NGQFFINGKPFYFKGFGKHEDTFINGRGFNEAANLMDLNLLKDIGAN<br>SFRTSHYPYSEEMMRLADRLGIVVLDEVPAVGLFQLFNAALNLTGDS<br>EEVKNTWEVMQTKEAHELVIDELIARDKNHPSVVMWVVANEPAGH<br>EKGARAYFEPLIQRMRDRDPSKRPVTLVNIMTATPDKDEVMDLVDV<br>VCLNRYYGWYVVHGDLNAAEKGLRQELETWQKLYPEKPILITEYGA<br>DTLPGLHSMWDIPYTEEFQVDYYDMNHRVFDSLPNLVGEQVWNFA<br>DFETTVGIIRIQGNHKGLFSRNRQPKQIVREIKKRWTAIPNYHYKRK
Bacillus sp. M4U3P1	A0A859FIK1	MLYPIFTETRGVLNLGGIWEFKLDDGRGLKDKWFEQKLTDTIPMAV<br>PASFNDIGVSGEIKNHVGDVWYETEITIPKVYKNERLVLRIGAATHK<br>ASVYVDGVFVAEHKGGFLPFEVELSSDSEKAKRRITIVVNNIIDDSTL<br>PVGVYEEEEVEGIGKVVRNMPNFDFFNYAGLQRPIKLYTTPRSYIKDI<br>ELVPQVTTDGGLLSYRVSTVLESVEEDYLVVAQLIDEEGKNVATGEG<br>LQGEMSVSDLRRWEPLNAYLYTFKATIMKGEQLIDTYEEPIGFRSVE<br>VRDGKFLINDKPFYFKGFGKHEDSPIAGRGFNEAVNVMDLRLMKW<br>MGANSFRTAHYPYSEELMRLADREGIVVIDETPAVGVHLNFSAASG<br>GFTKEDSTWSRIKTFDHHKDVLNDMIARDKNHACVVMWSVANEAA<br>TEEEGAYEYFKPLVELTRERDPQSRPVMIVTFIKSTPELDKIADLIDVL<br>GFNRYYGWYENSGELDVAKVRLAAELAAWEKRCPGKPMMMTEYG<br>ADTIAGYHEIVPTMFTEEYQSDYLKINHEVFDQVPTFIGEHVWNFAD<br>FATSQNVRRVNGNKKGVFTRERKPKNAAHELRKRWTAIDDFYYKT
Escherichia coli (strain K12)	P05804	MLRPVETPTREIKKLDGLWAFSLDRENCGIDQRWWESALQESRAIAV<br>PGSFNDQFADADIRNYAGNVWYQREVFIPKGWAGQRIVLRFDAVTH<br>YGKVWVNNQEVMEHQGGYTPFEADVTPYVIAGKSVRITVCVNNEL<br>NWQTIPPGMVITDENGKKKQSYFHDFFNYAGIHRSVMLYTTPNTWV<br>DDITVVTHVAQDCNHASVDWQVVANGDVSVELRDADQQVVATGQ<br>GTSGTLQVVNPHLWQPGEGYLYELCVTAKSQTECDIYPLRVGIRSVA<br>VKGEQFLINHKPFYFTGFGRHEDADLRGKGFDNVLMVHDHALMDW

		IGANSYRTSHYPYAEEMLDWADEHGIVVIDETAAVGFNLSLGIGFEA<br>GNKPKELYSEEAVNGETQQAHLQAIKELIARDKNHPSVVMWSIANE<br>PDTRPQGAREYFAPLAEATRKLDPTRPITCVNVMFCDAHTDTISDLF<br>DVLCLNRYYGWYVQSGDLETAEKVLEKELLAWQEKLHQPIIITEYG<br>VDTLAGLHSMYTDMWSEEYQCAWLDMYHRVFDRVSAVVGEQVW<br>NFADFATSQGILRVGGNKKGIFTRDRKPKSAAFLLQKRWTGMNFGE<br>KPQQGGKQ
Sphingomonas aquatilis NBRC 16722	A0A511PZJ0	MLAAALFVLPTGGAAQQPTQAIAGEGWPVLAAADLRAGVDLDGA<br>WHYSVDPYRAGLSGFHGGAPGLSDRRWADVDVGETMRRDTRAAFE<br>FDMDRAPVATLPGSWLTHAAELRHYQGLMWYQRHVVVHPTPGKR<br>VFLRFGAVNYTARVFLNGRPVGQHEGGFTPFAFDVTAALRDGDNQI<br>TVGVDSTATPASVPPPVTDWETYGGITRSVRLIETPETYVDDAWVRL<br>TRTGRIAADIHLDGQRAGGQAVTLGIAALGVRIRGRTDAAGNWHAE<br>LPVPRSLVRWSPDRPQLYDVTVTAGDDRWRDRVGFRTLAVRGATLL<br>LNDRPIFLRGISLHEEEFGPNPARIMTRAASRALLMEAKTGLHANYV<br>RLAHYPHAEVTTRLADELGLLVWSEVPVYWLIDWANPATLATARR<br>MVADNVRRDRNRASIAIWSVANETPVTDARNSFLGTLVGDIRALDD<br>TRLLSAALLVKRTTEAGRPVMTLDDPLAATLDVMAVNTYTGWYGN<br>DALSVVPKTLWRVPADRPLVLSEFGAGAKAGFHDTRAAPQKFSEEF<br>QAAYYRATLAMTEHMPTLAGLSPWILKDFRSPRRQNGFQQGWNRK<br>GLVSEKGQRKQAFAVLAEYYAARDVASCTGRVGAEHAPC
Citrobacter amalonaticus	A0A6N2X9S7	MLEKSDLADNAVECLHNEHYDRAYNVQNLNFRTLIFSGGRQRENLA<br>GDWRFTLDLHDTGLRQKWFLMQKQDAETRKDPYDYDPYSGQTVPV<br>PSCWQMLKDKWFYYEGSAWYTREIEYAKRDPDERVFLRIGAANYD<br>CKIFLNHQFIGNHYGGSTPFCAELSTPIKAGNNVLMICVNNTRTTDRL<br>PLRNNDWFNYGGIYREIELIRTPRNYIHDLFVYLVPDGNFNRIAARIT<br>VDGPATDVTLMIPELGINQILPVVDGQAEVILSVSPELWSPENPKRYK<br>VRSMLGRDSVEDDVGFRQIEVVGTDIRLNGKSIYLRGVNCHEDDLF<br>MGKVTCEDDIRQRFRDAKALNCNYMRLAHYPHHELAARIADEEGIL<br>LWEEIPNYWAVDFTNPSTQRDAHNQLMELILRDRNRASVIIWSVGNE<br>NADTDERLSFMTSLVNAARDADPSRLISAACLVNHAKMKIEDRLTE

		YLDIIGLNEYYGWYEENFDDLILLGKNSSPTKPVVITETGAEGVISED<br>APKTGPFSEQYMAEVYRKQTEYLPKLTYVRGFTPWLLYDYRTERRQ<br>NPWQQGVNCKGLITADKKTRKAAFYVLRDFYENLKKVESSSD
Enterococcus gallinarum 1	A0A376H732	MSRNEYPRPQFQRENWLNLNGEWHFAFDDKNVGLKEKWYQEEEA<br>YPHRIKVPFVYQSELSGINQRESHDIVWYYRTFTVQAMDSQRVVLHF<br>GAVDYEADVFVNGCHVTNHQGGHTSFEVDITDYLVDGQQAISVRVF<br>DPHADESIPRGKQFWETESAGIWYTNSTGIWQPVWLEVVSETYLKEI<br>NLTPNFDEGTVTVETILNQFQPQCELDYRISFKEQLIAAGRLSADSAK<br>MKFNVDLFQEHIFRSNFHHDGWSWTPENPRLFDIKLSLINDGETIDYL<br>KSYFGMRKIHTENGMVYLNNKPYYQKLILDQGYWPSGLLTAPSDED<br>FKKDILLAKEMAFNGCRKHQKTEDPRFLYWADQLGFLVWVECAAP<br>AIYNEDSVERLMREWTEIIARDFSHPSIVTWVPINESWGVPKISFDRT<br>QQHFSQAMYHFLHALDKTRLVISNDGWAMTETDICAIHNYAHGQKE<br>ETEKYDYFKETLRTRENLIKRLSTPWPIFANGFSYQEQPILLTEFGGIG<br>FDVSGQPGWGYTSVDNEVEFLKDYQRVLSAVYASVGLWGYCYTQL<br>TDVEQEINGLLTYDRQPKVDLAAIKAINDQFHVSQVE
Enterococcus gallinarum 2	A0A376GWX3	MLYPIMTETRQVIDLSGLWRFKLDTNDDLQEELALEPINEKESYPISV<br>PSSYNDLFETENIRNHVGWVWYEKEVTLPKRLLEERLVLRFGSATHE<br>AKVFLNGKLITQHKGGFTPFESEINRFVKDGNNRITVAVNNIIDDSTL<br>PVGIMKETVQENGDIKKENLVNFDFFNYAGIHRPVKLYTTPKDYISDI<br>TINTRITDLDASVEFNIDTEWTGKILIAIYDEEAQLVAEAEGKKGMIQ<br>LKNPIFWEPLDAYLYSMKISLIDDNKVIDTYTEEFGIRSVQVTNGKFLI<br>NNKPFYFKGFGKHEDSYVNGRGLNEAVNIKDFNLMKWIGANSFRTS<br>HYPYSEEIMRLADREGIVVIDEVPAVGLHLNFMATLLDDNVEKHNT<br>WKEIRTEDSHKTVIKELIERDKNHPSVVMWSIANEPDSDSEGAKEYF<br>EPLVELTKELDPQKRPVTIVTYLKSTPDVCKVGDIVDVLCLNRYYGW<br>YVSGGDLSHAKFLLKKELDGWMERCPDTPIIFTEYGADTVAGFHDT<br>VPTMFTEEYQVAYYEANHEVIDTCENFVGEQVWNFADFATSQGILR<br>VQGNKKGIFTRDRKPKMIAYYLQKRWSSIPNFSYKK
		MLRPVETQSREIKKLDGLWSFCTDADDCGIAQQWWRQPLPQSRAIA<br>VPGSYNDQFADAEIRNYVGNVWYQLNVRIPKGWEHQRIVLRFDAVT<br>HYGKVWVNDHAVMEHQGGYTPFEADISHLAAAGESIRITVCVNNEL

Salmonella enterica	A0A5I9F3M6	SWQTIPPGVVTRDETGKRQQSYFHDFFNYAGIHRSVMLYTTPKTFVQ<br>DITVTTEVANDLSRATLSWRVEANGEVRVELRDAGQHIVAYGEGAK<br>GELQIAAPRLWQPGEGYLYELRVIAQHQDERDDYPLRVGIRSVAVK<br>GEQFLINHKPFYFTGFGRHEDADLRGKGFDNVLMVHDHALMSWIGA<br>NSYRTSHYPYAEEMLDWADEHGIVVIDETAAVGFNLSLGIGFDSCEK<br>PKALYSEEGVNDETQRAHLKAIQELIARDKNHPSVVMWSIANEPDTR<br>PEGARDYFAPLAQATRELDPTRPITCVNVMFCDVETDTITDLFDVVC<br>LNRYYGWYVQSGDLAKAEKVLEKELLAWQEKLHRPIIMTEYGVDT<br>LAGLHSMYSDMWSEEYQCAWLEMYHRVFDRVSAVVGEQVWNFA<br>DFATSQGIMRVGGNRKGIFTRDRRPKSAAFILQKRWTGMTFGEKPQ<br>QGEKL
Staphylococcus caeli	A0A1D4IY47	MLYPVVNDYRSIIDLNGIWQFKLENNNETVDVSQPLQTDKVMAVPG<br>SYNDQGVTANIRNHVGNVWYERTFTVPNVLRNERIVLRFGSATHKA<br>TIYIDGQEVTSHQGGFLPFEVELSEVHSSGTHRLTVCVSNILDETTLPV<br>GDYSESTDKDGNLIKKNSPNFDFFNYAGLHRPVKIYTTPQTFIEDIEIV<br>PELEADIALVKYKVTTSAQVEAVQVRLVDEDDNYIAEATGESGTIQV<br>EHPHLWEPLNAYLYHLEVSILENGTVIDTYAERFGIRSVEVKNGQFLI<br>NDKPFYFKGFGKHEDSYYNGRGMNEVTNVLDFNLMKWIGANSFRT<br>SHYPYSEEMMRLADEQGIVIIDETTAVGVHLNFSAILSGNRTRDTFKE<br>IGTKAAHEDVIKGLIARDKNHACVVMWSIANEPASDEVGAKEYFEPL<br>INLARTSDPQNRPVTIVTILTSQPDTCQVQDLVDVLCLNRYYGWYTQ<br>SGDLEAAKIALEQELDGWTEKQPGKPIMFTEYGADTVAGMHSLNDE<br>LFTEEYQIHYYEANHEVIDRYPQFIGEQTWNFADFETSTGIIRVQGNK<br>KGIFTRERRPKAVAHYFKQRWESIPDFGYKG
Staphylococcus haemolyticus	A0A8F3PTH5	MLYPINTETRQLLDVSGIWKFKLVGYKEHVDVSQALDTEDVMAVPG<br>SYNDQGVIQSIRQHVGDVYYEREIMIPKHLKGERIVLRFGSVTHHAT<br>VYLDGKEIVTHSGGFLPFEVNITEIATTGSHRLTVKVNNILTHSTLPVG<br>NYSERVDEDGTVTPVNTPNFDFFNYAGIHRPVKIYTTPQTYIQDIVINP<br>EVKQSGESIVNYKVNVHGNRSDIDRISVTIIDEDGQVVTKHDESEGA<br>MSIDNPHLWQPLNAYLYHMKVELLNDGEVVDVYTERFGIRSVEVKD<br>GQFLINNKPFYFKGFGKHEDTFYSGRGLNEAANVMDINLMKWIGAN<br>SFRTAHYPYSEEMMRLADEQGIVVIDETTAVGVHLNFMVALGGQLE

		HDTWKEIDTHQAHKEVIEGLIERDKNHACVVMWSIANEPASNEKGA KAYFEPFVELAKRKDPQKRPVTIVTILMAQPDVCEVQDLVDVLCLNR YYGWYTQSADLASAKKALDKELAQWSERQPNKPIMFTEYGADTVA GMHAIDDQMFTEEYQLNYYKANHEIMDKYPQFIGEQTWNFADFETS NGIIRVQGNKKGIFTRDRKPKAIAHYFKERWHNIPDFNYKS

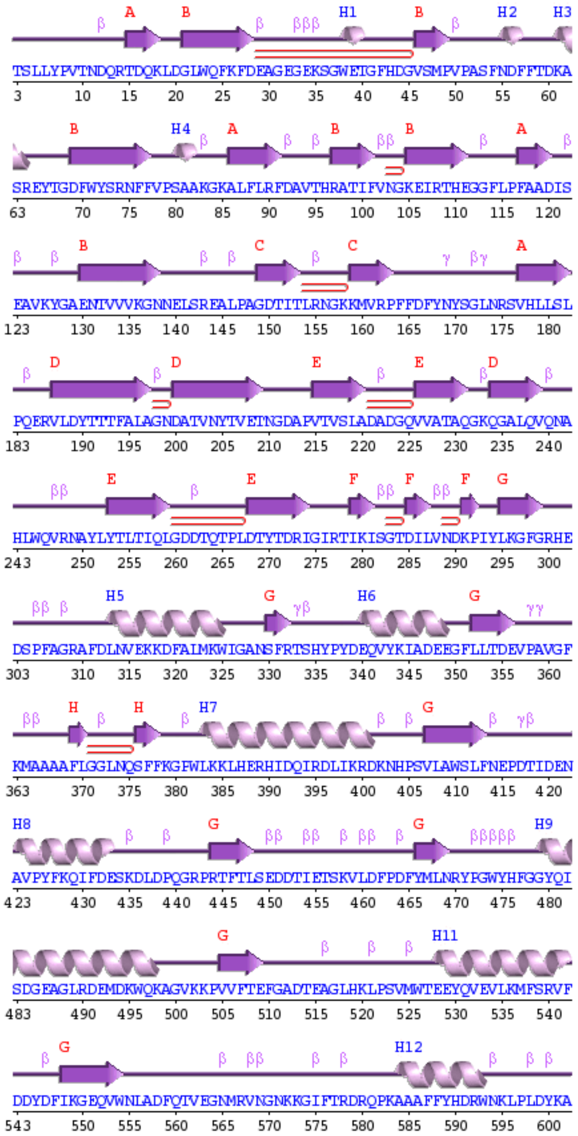

(a)

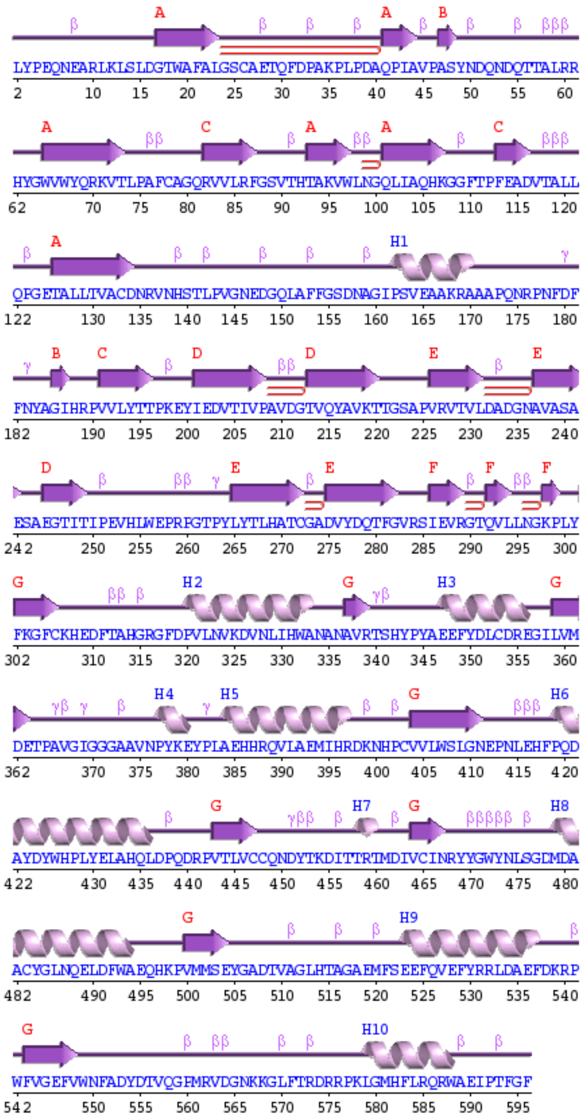

(b)

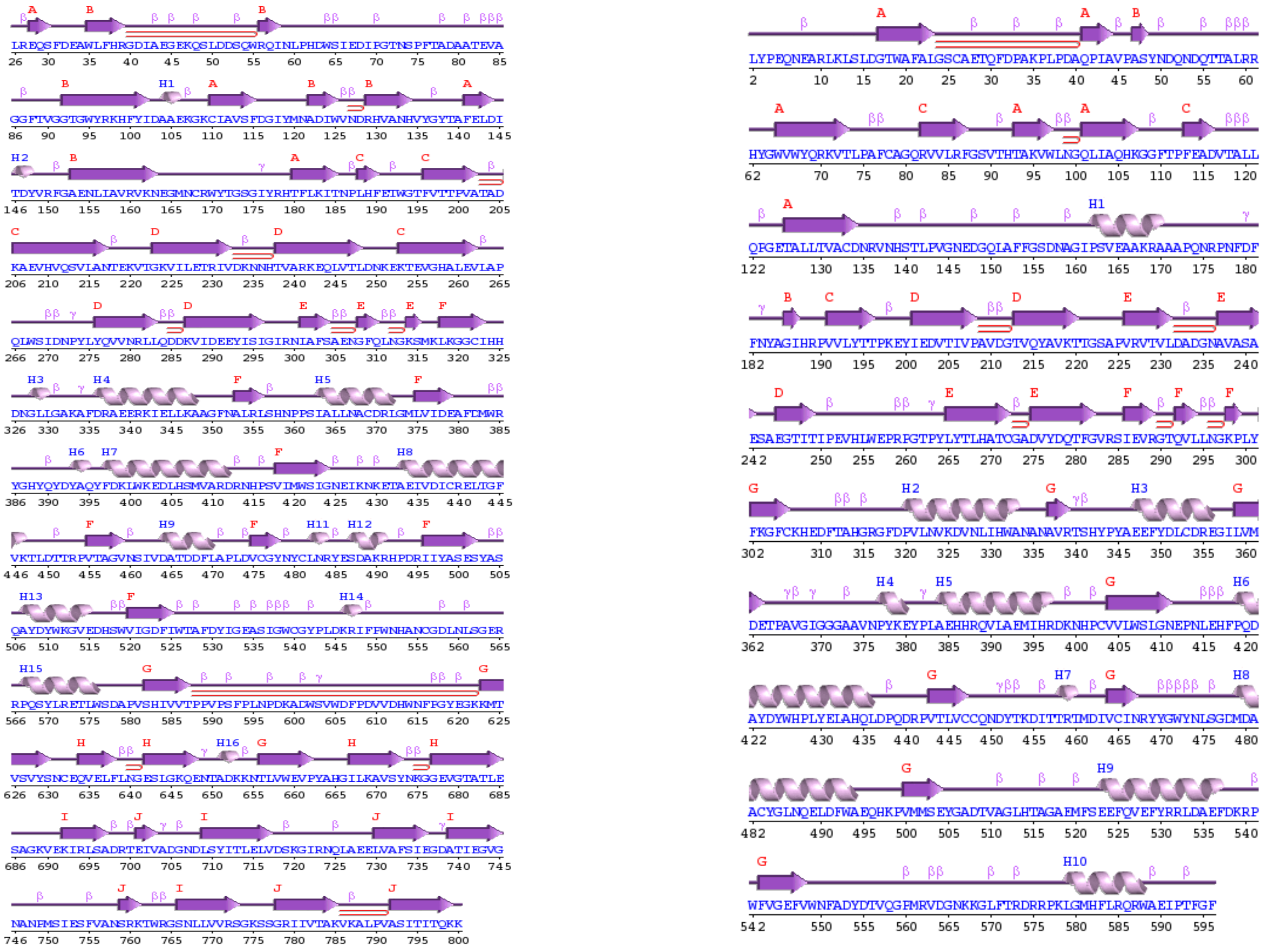

(c)

(d)

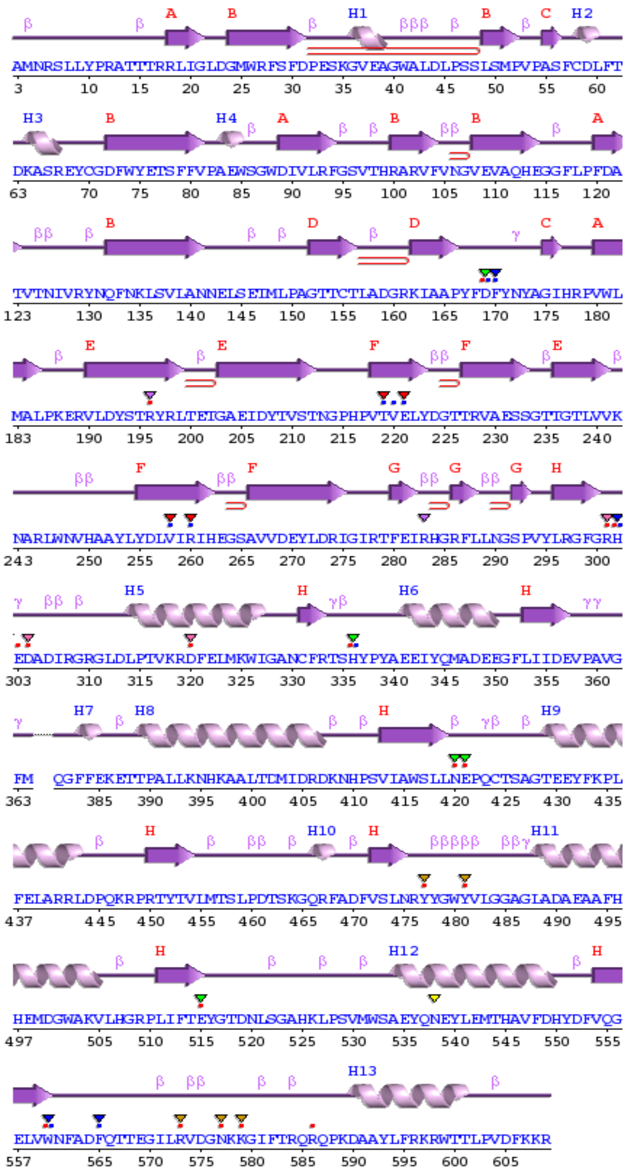

(e)

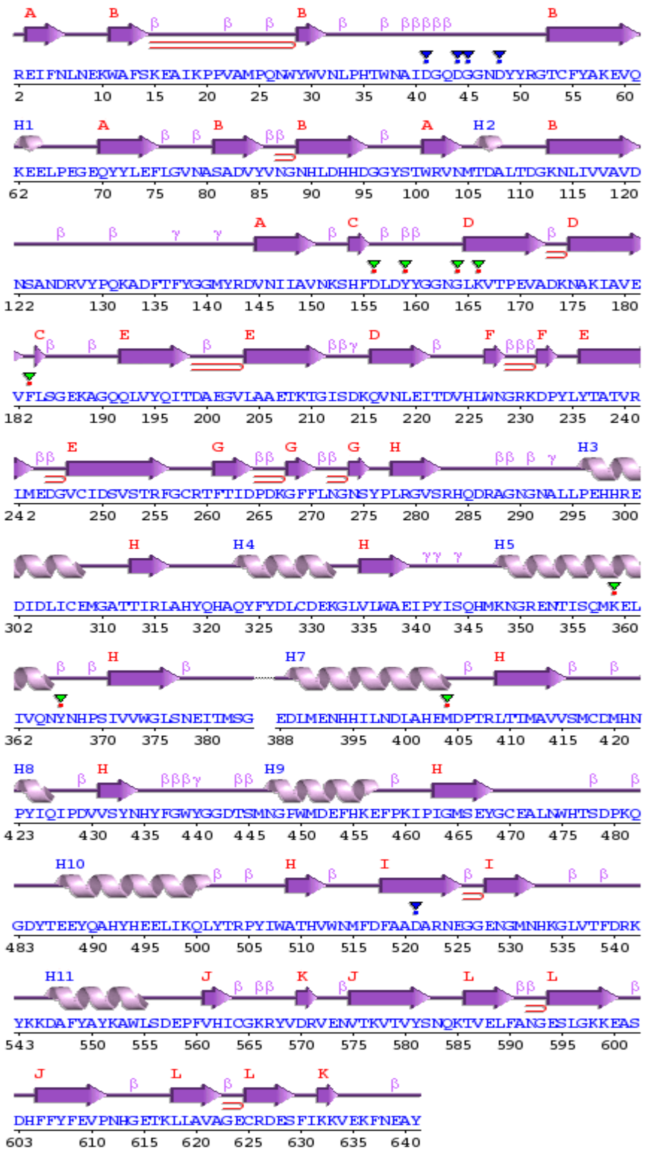

(f)

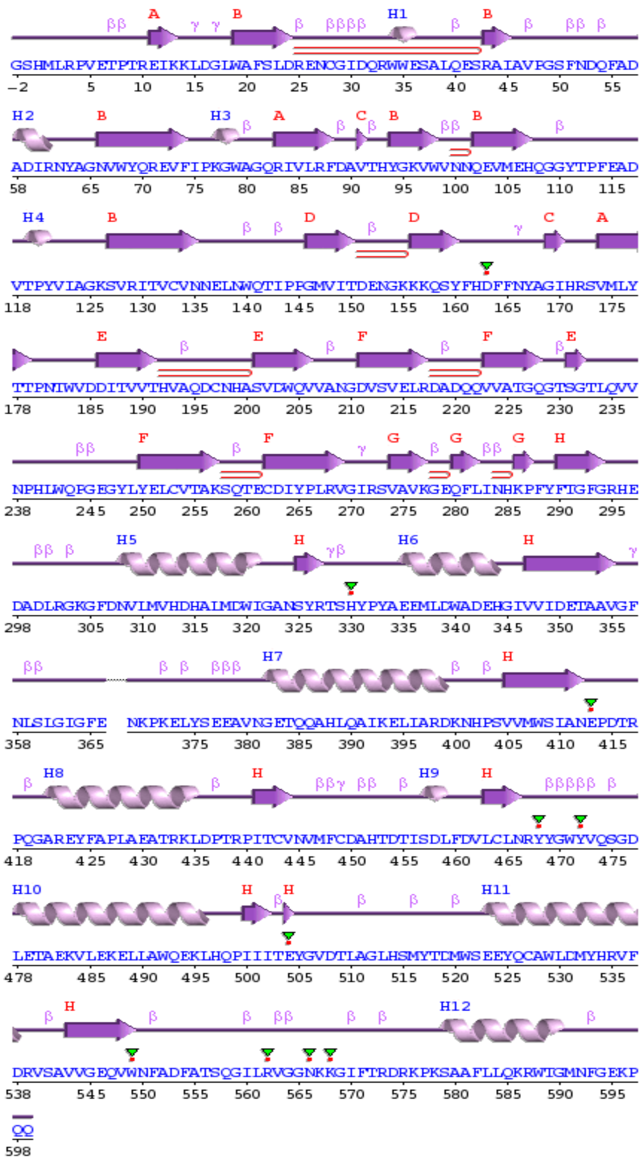

(g)

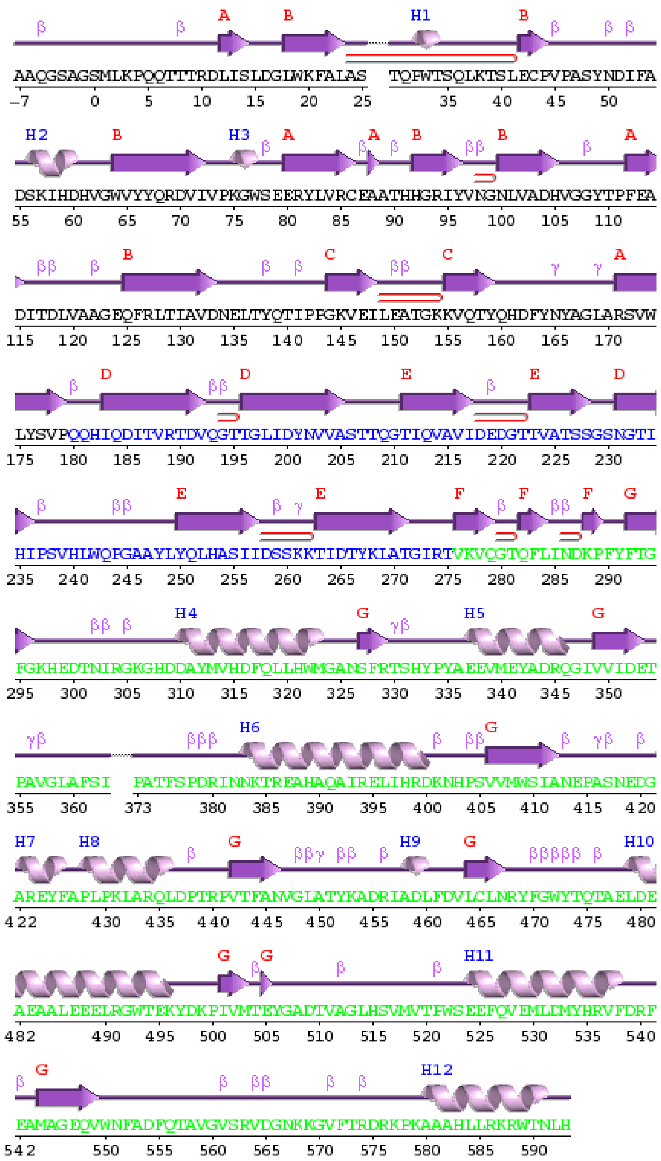

(h)

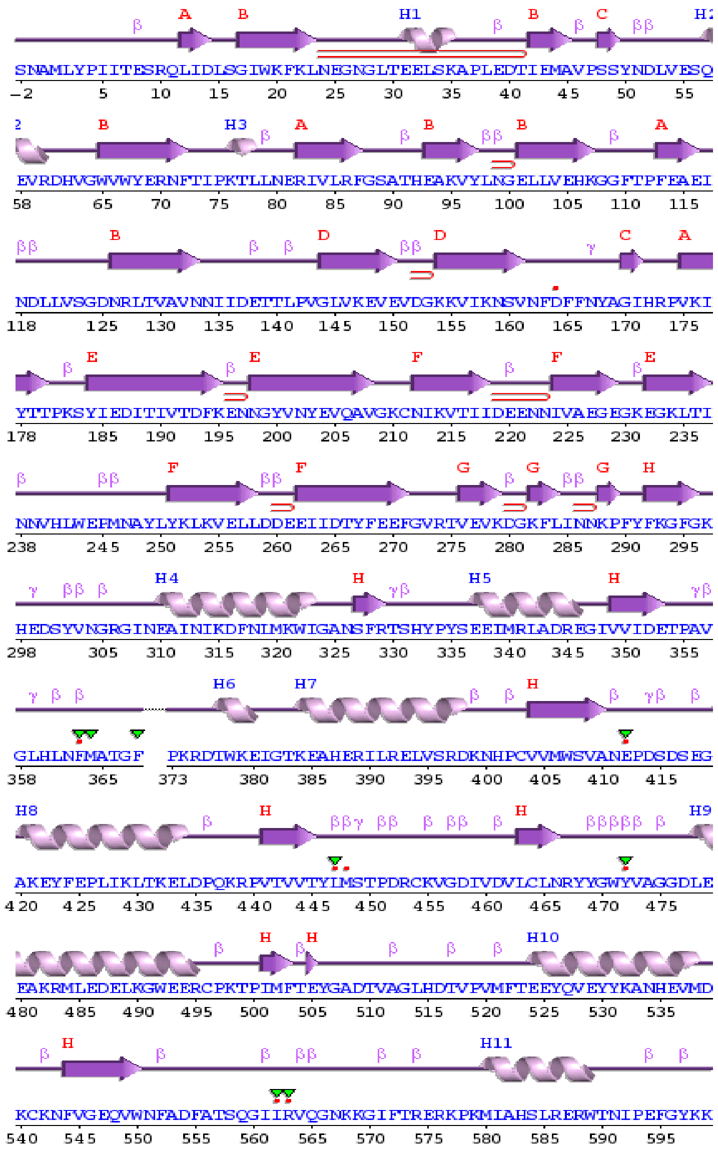

(i)

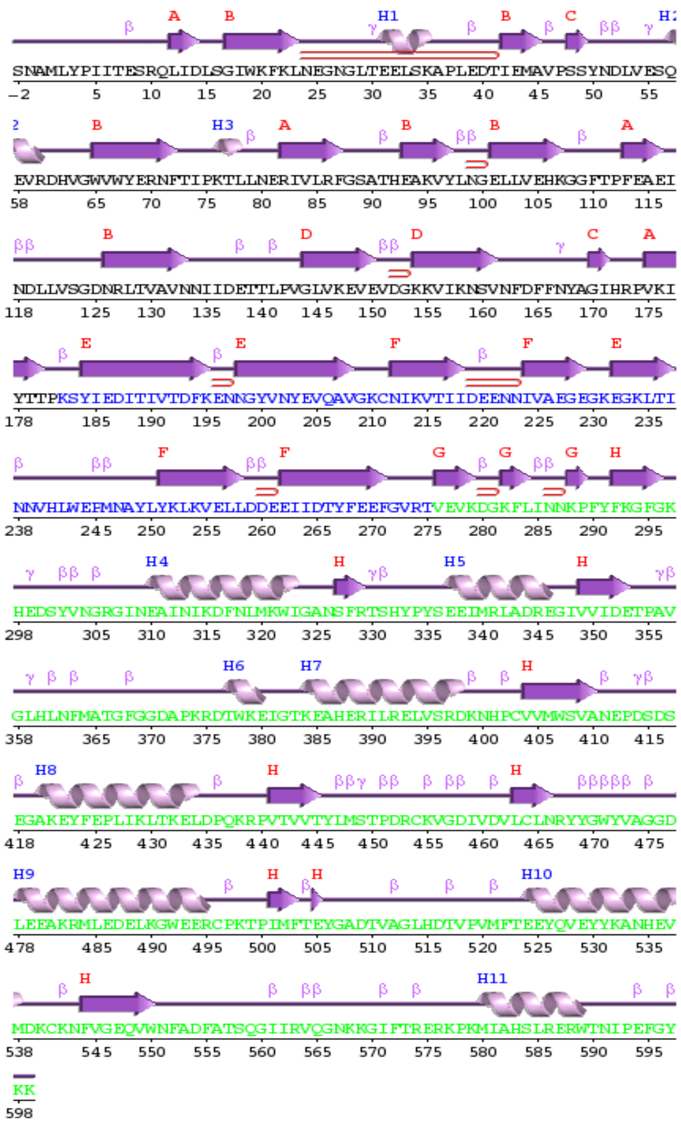

(j)

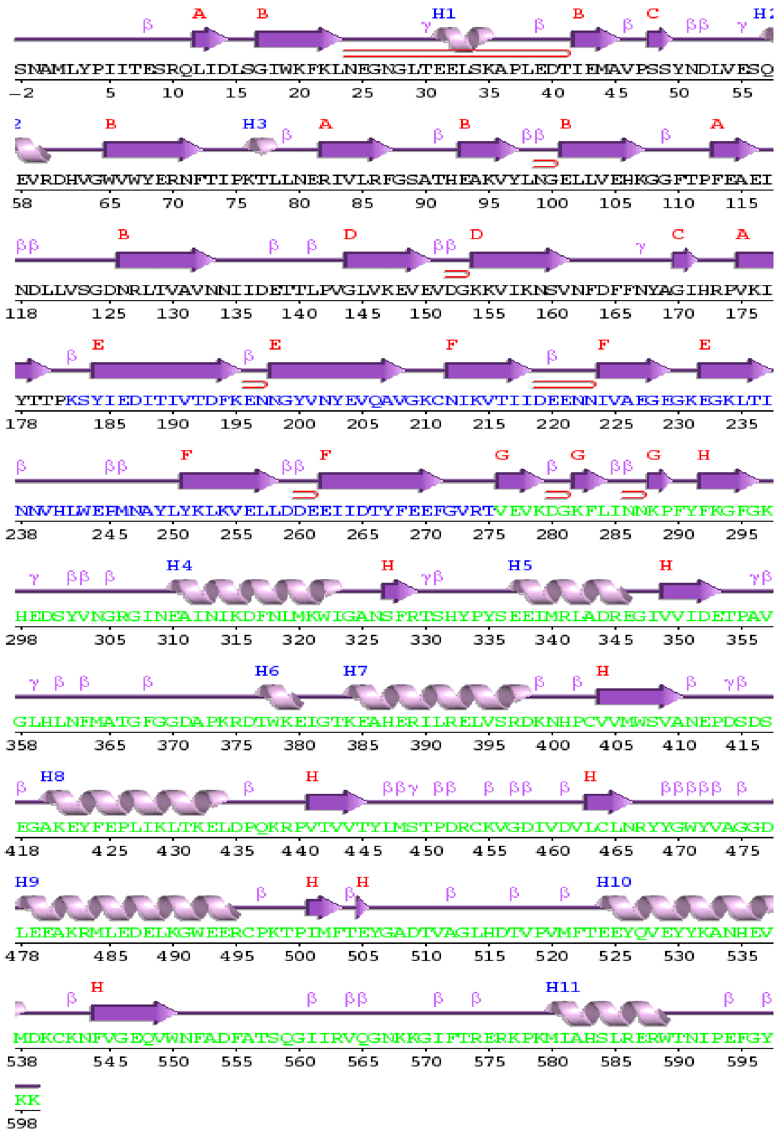

(k)

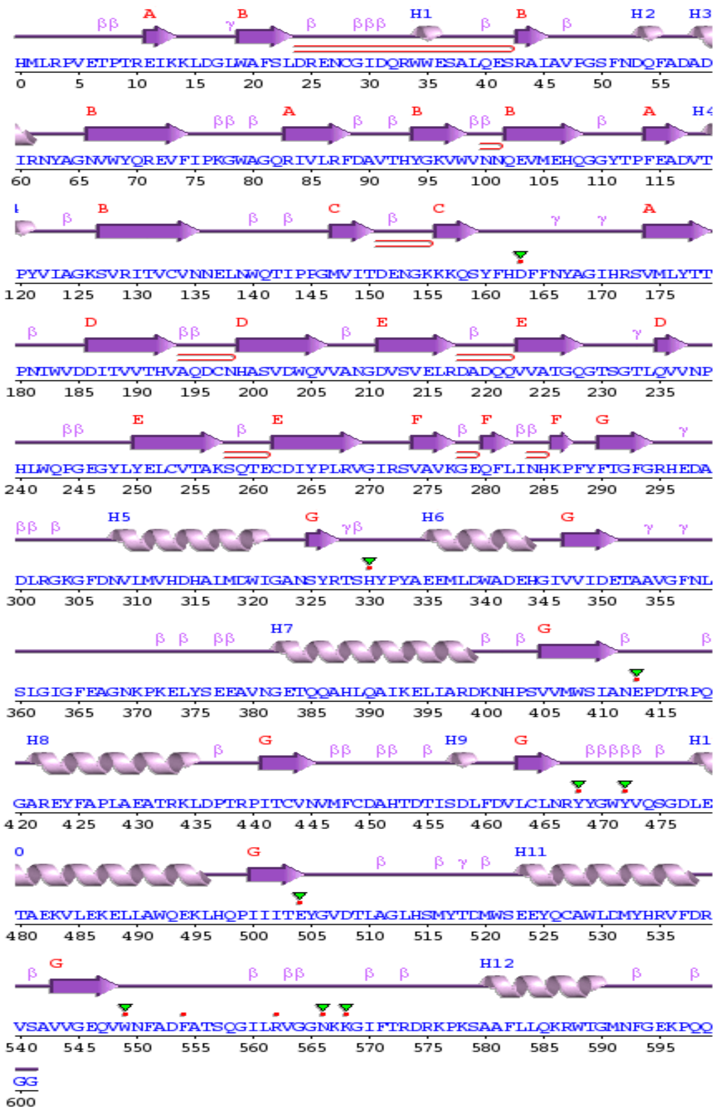

(l)

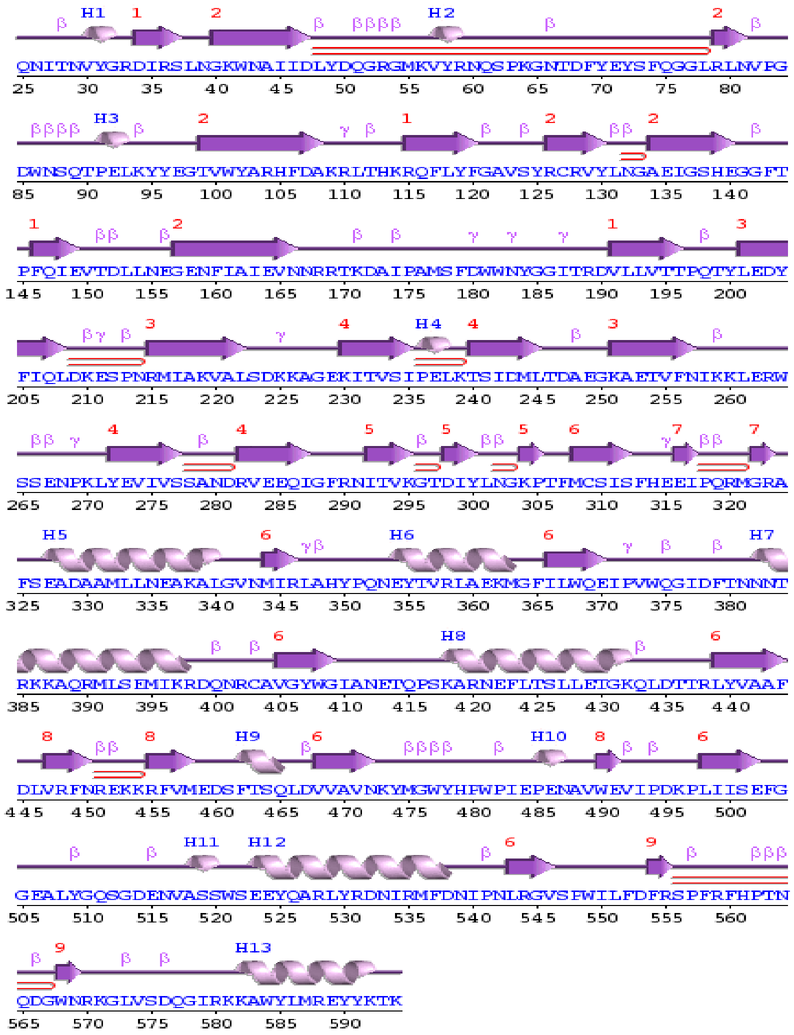

(m)

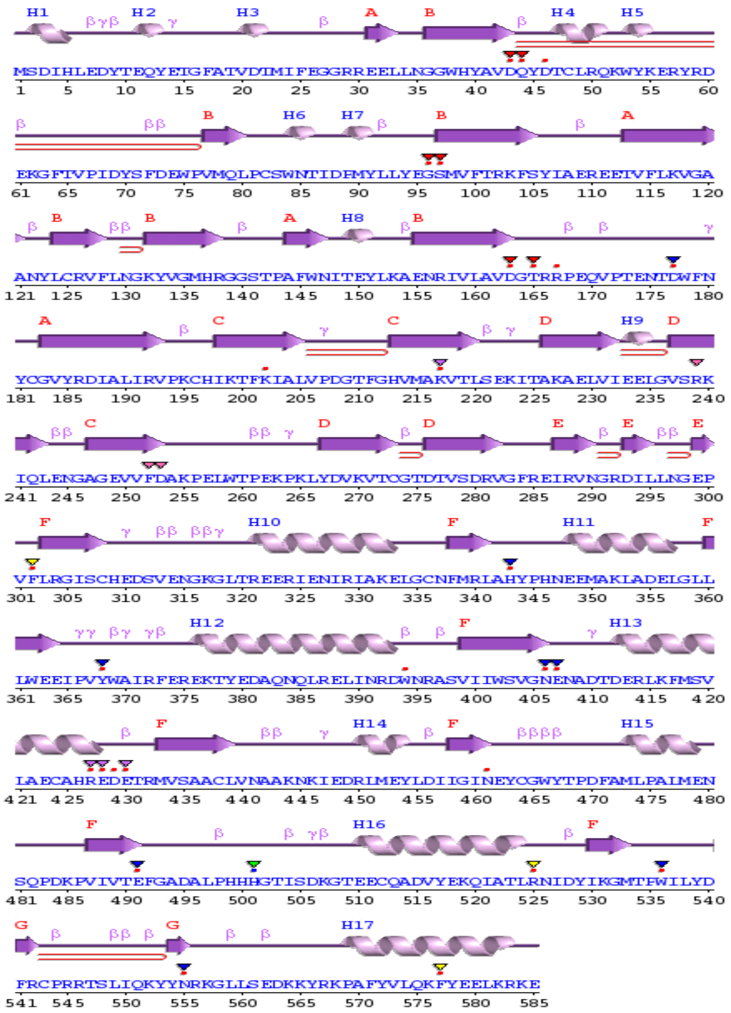

(n)

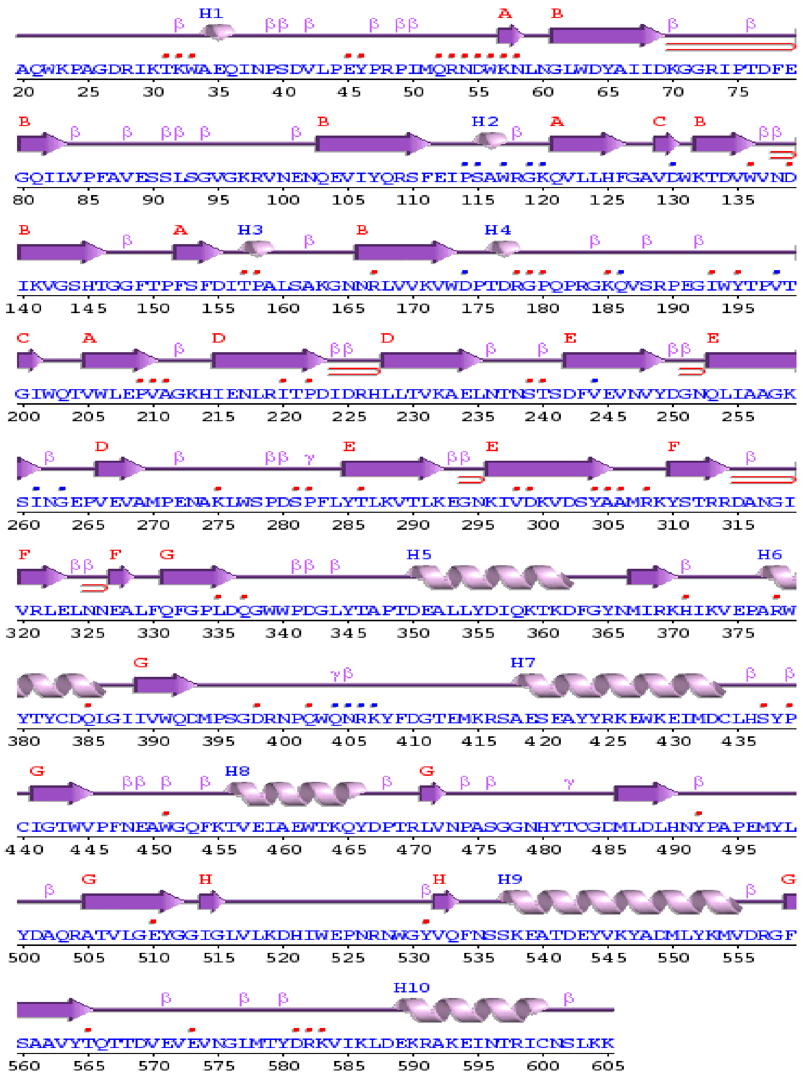

(o)

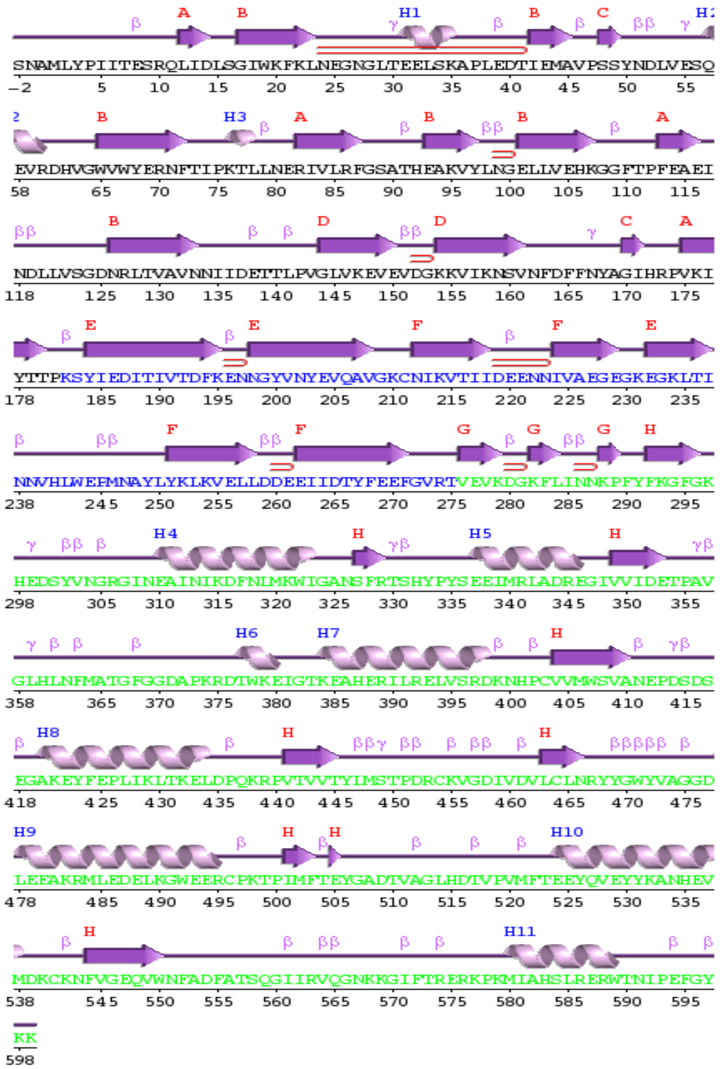

(p)

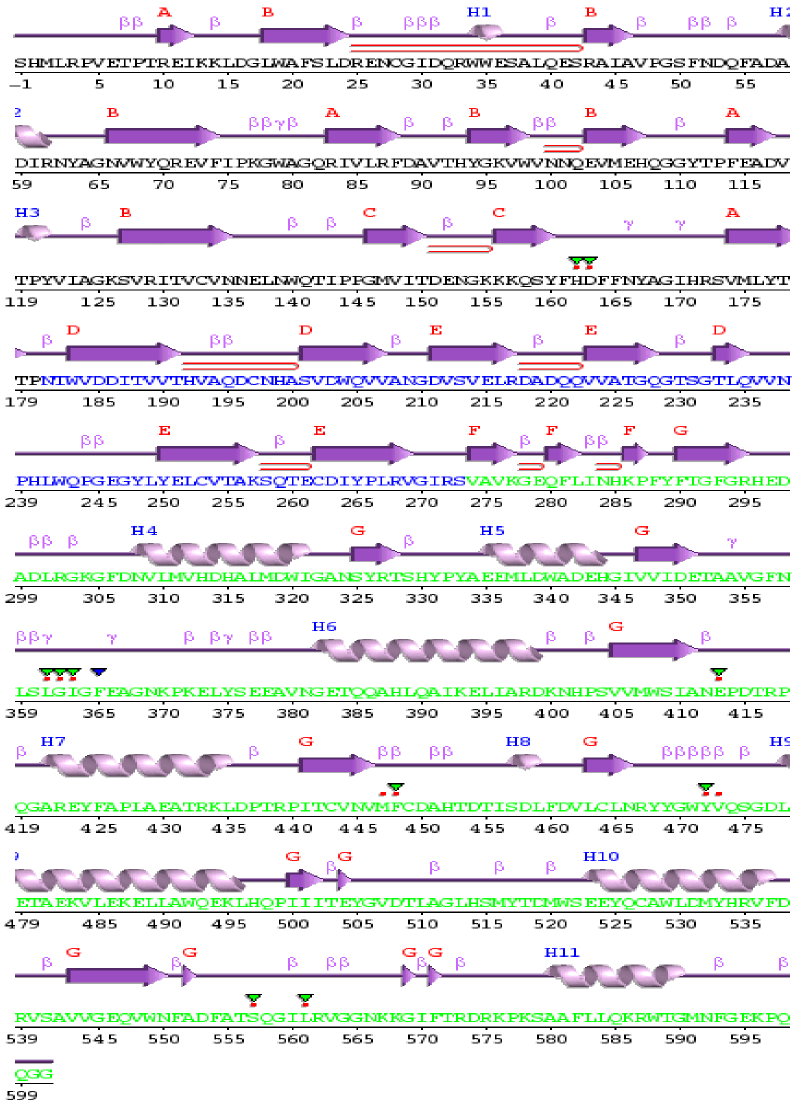

(q)

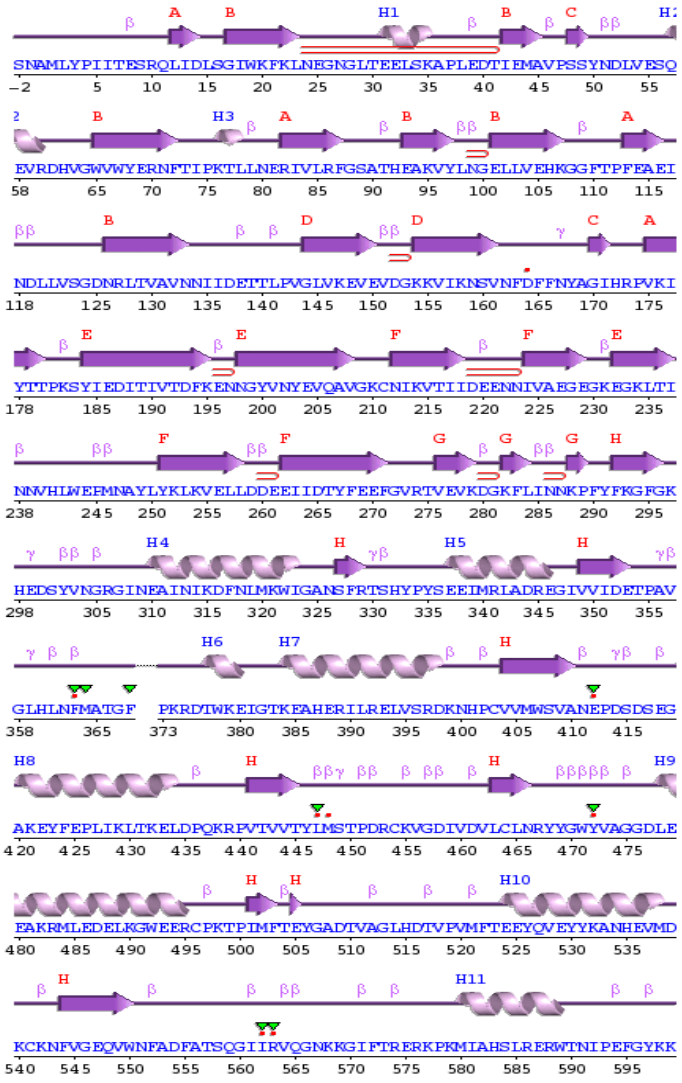

(r)

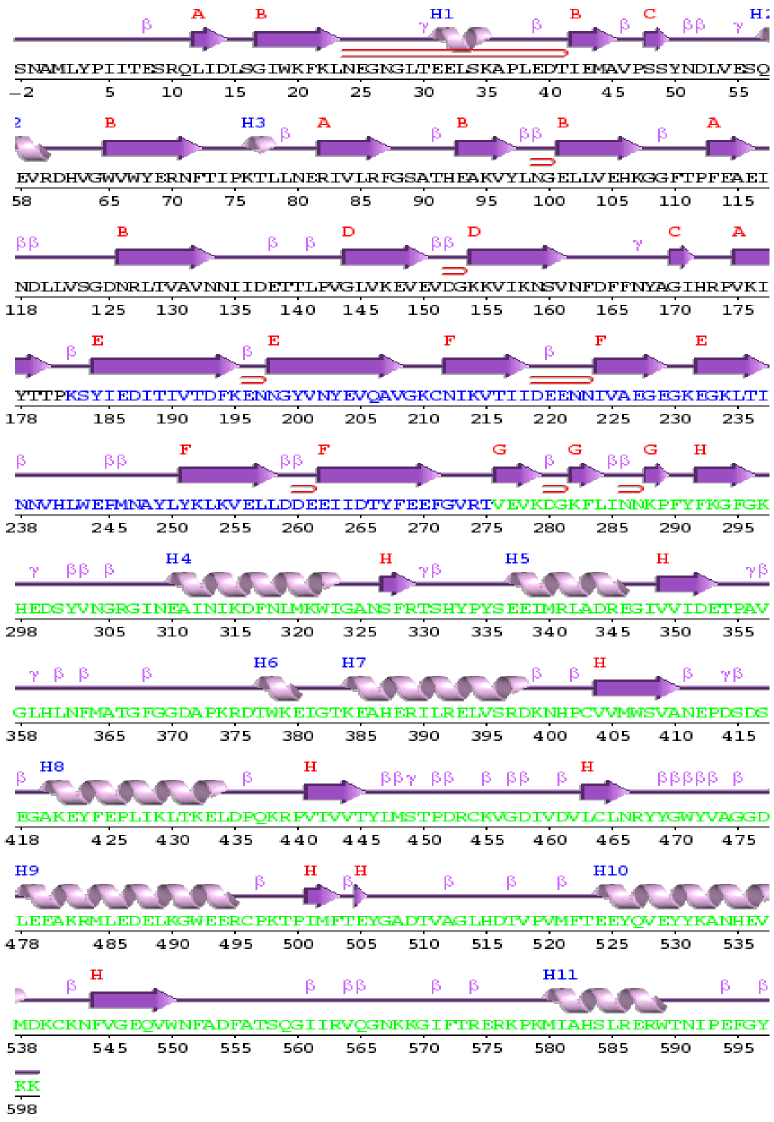

(s)

Supplementary Data Figure S1: Secondary structure motif maps of GUS enzymes for probiotics predicted by PDBsum tool

(a) *L. rhamnosus*, (b) *R. intestinalis*, (c) *C. comes*, (d) *F. prausnitzii* 1, (e) *F. prausnitzii* 2, (f) *F. prausnitzii* 3, (g) *P. acnes*, (h) *M. bacterium*, (i) *S. xylosus*, (j) *S. suis*, (k) *Bacillus* sp. M4U3P1, (l) *E. coli* (strain K12), (m) *S. aquatilis* NBRC 16722, (n) *C. amalonaticus*, (o) *E. gallinarum* 1, (p) *E. gallinarum* 2, (q) *S. enterica*, (r) *S. caeli*, (s) *S. haemolyticus*

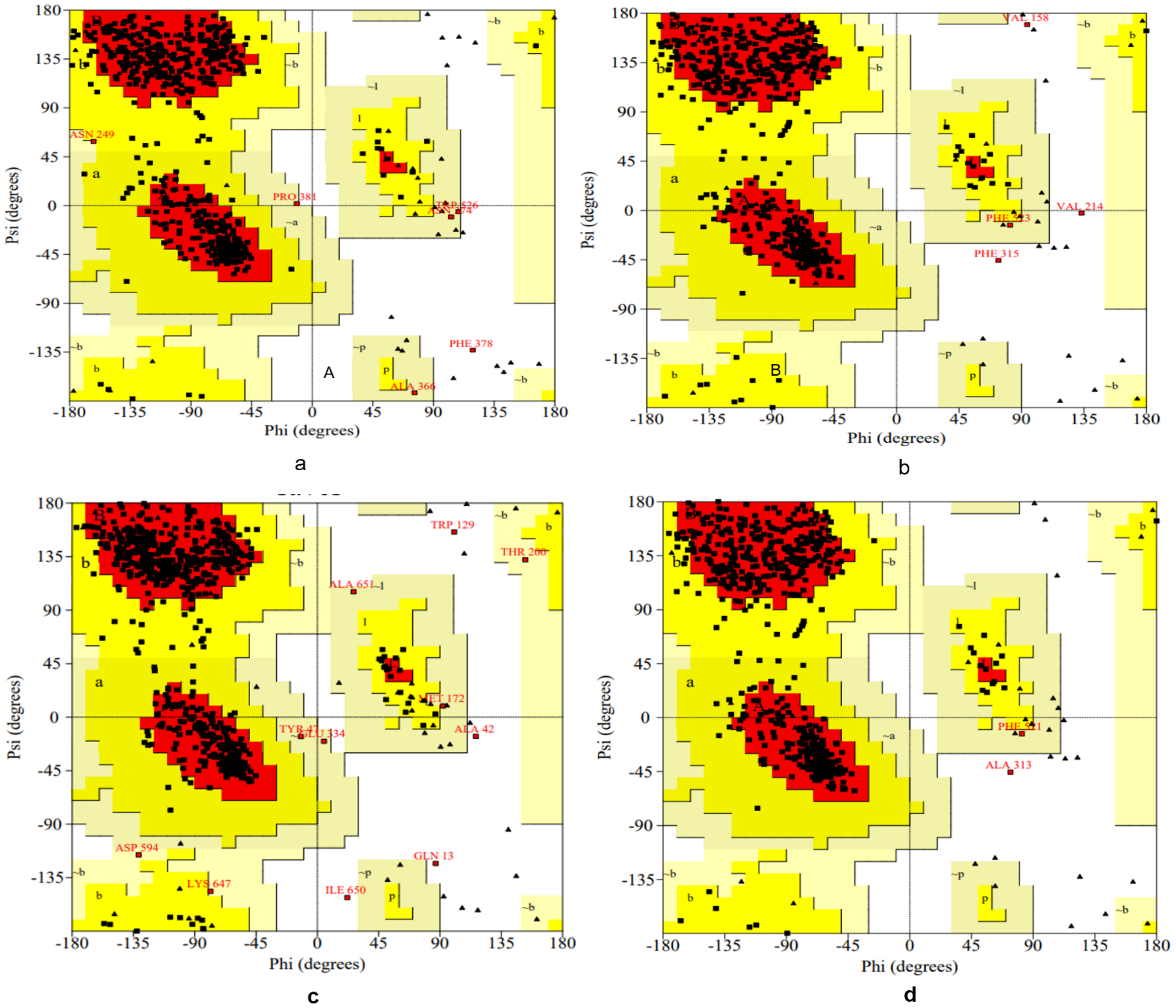
Psi (degrees)
Phi (degrees)
ASN 249
PRO 381
PHE 378
ALA 366
VAL 158
VAL 214
PHE 315
TRP 129
THR 200
ALA 651
MET 172
ALA 42
ASP 594
LYS 647
ILE 650
GLN 13
ALA 313
A
B
a
b
c
d

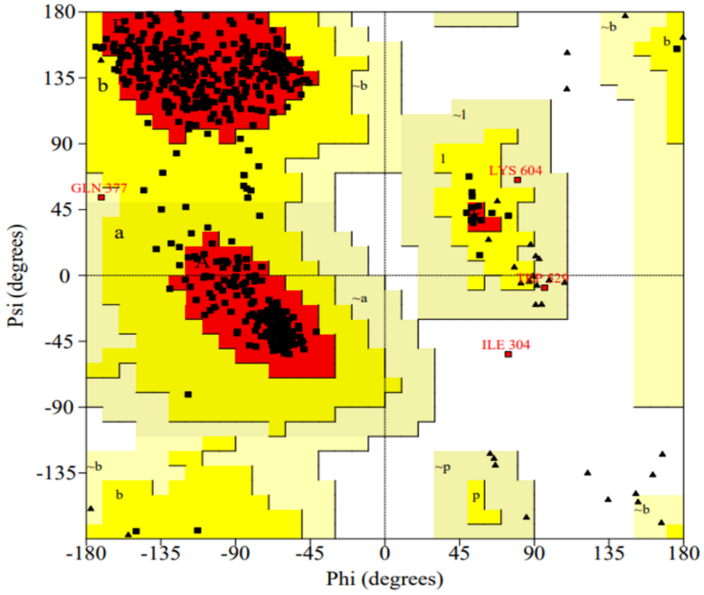

Psi (degrees)
Phi (degrees)
GLN 377
LYS 604
TRP 529
ILE 304

e

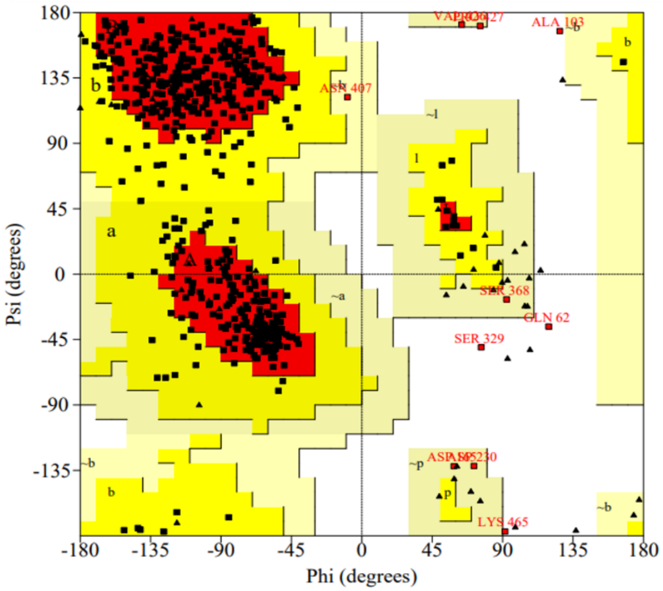

Psi (degrees)
Phi (degrees)
ALA 103
ASN 407
SER 368
GLN 62
SER 329
LYS 465

f

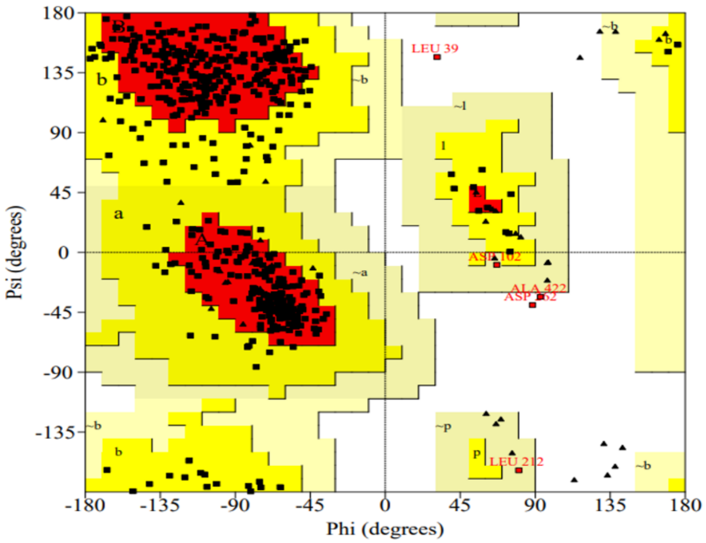

Psi (degrees)
Phi (degrees)
LEU 39
ASP 102
ALA 422
ASP 62
LEU 212

g

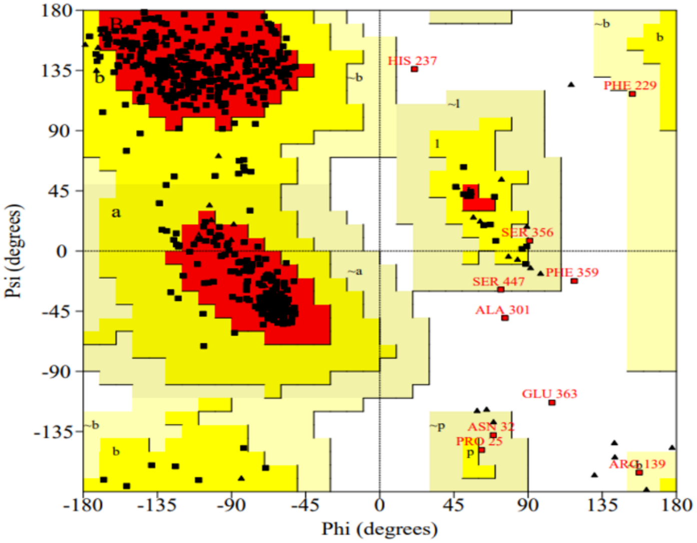

Psi (degrees)
Phi (degrees)
HIS 237
PHE 229
SER 356
PHE 359
SER 447
ALA 301
GLU 363
ASN 32
PRO 25
ARG 139

h

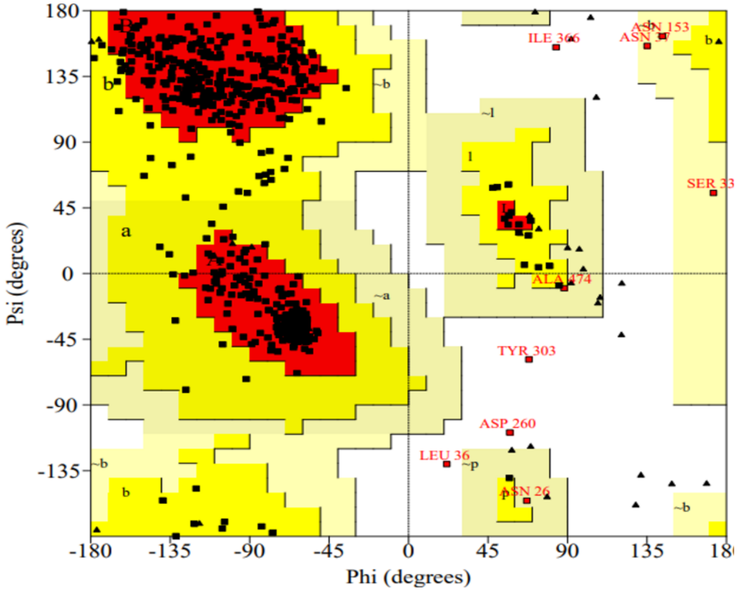
Psi (degrees)
Phi (degrees)
ILE 366
ASN 153
SER 33
ALA 174
TYR 303
ASP 260
LEU 36
ASN 26

i

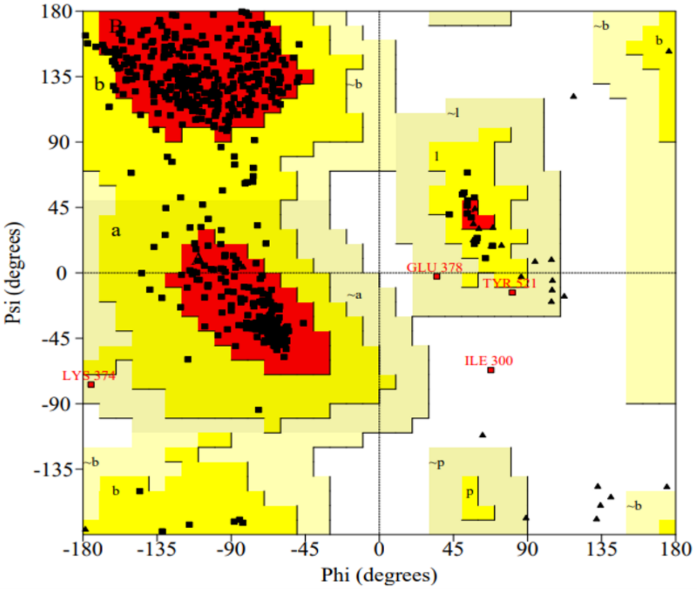
Psi (degrees)
Phi (degrees)
GLU 378
ILE 300

j

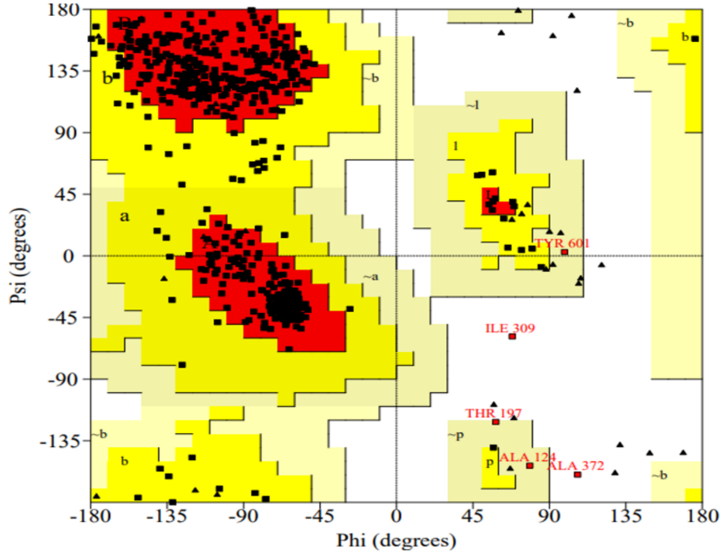
Psi (degrees)
Phi (degrees)
TYR 601
ILE 309
THR 197
ALA 372

k

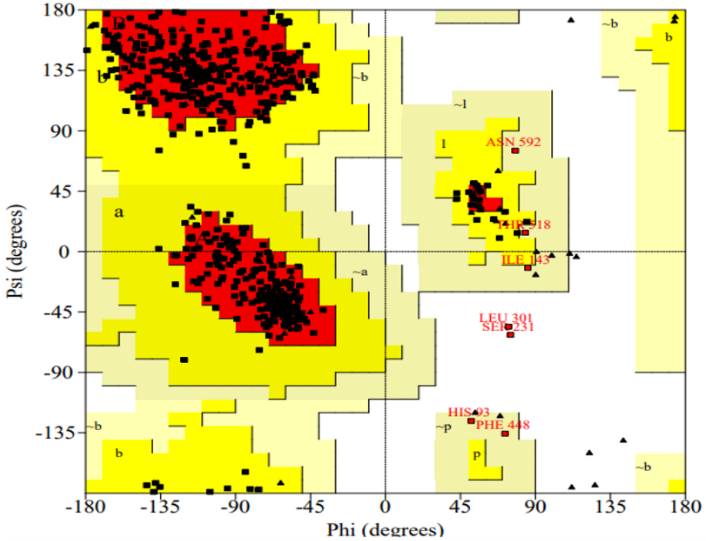
Psi (degrees)
Phi (degrees)
ASN 592
PHE 448

l

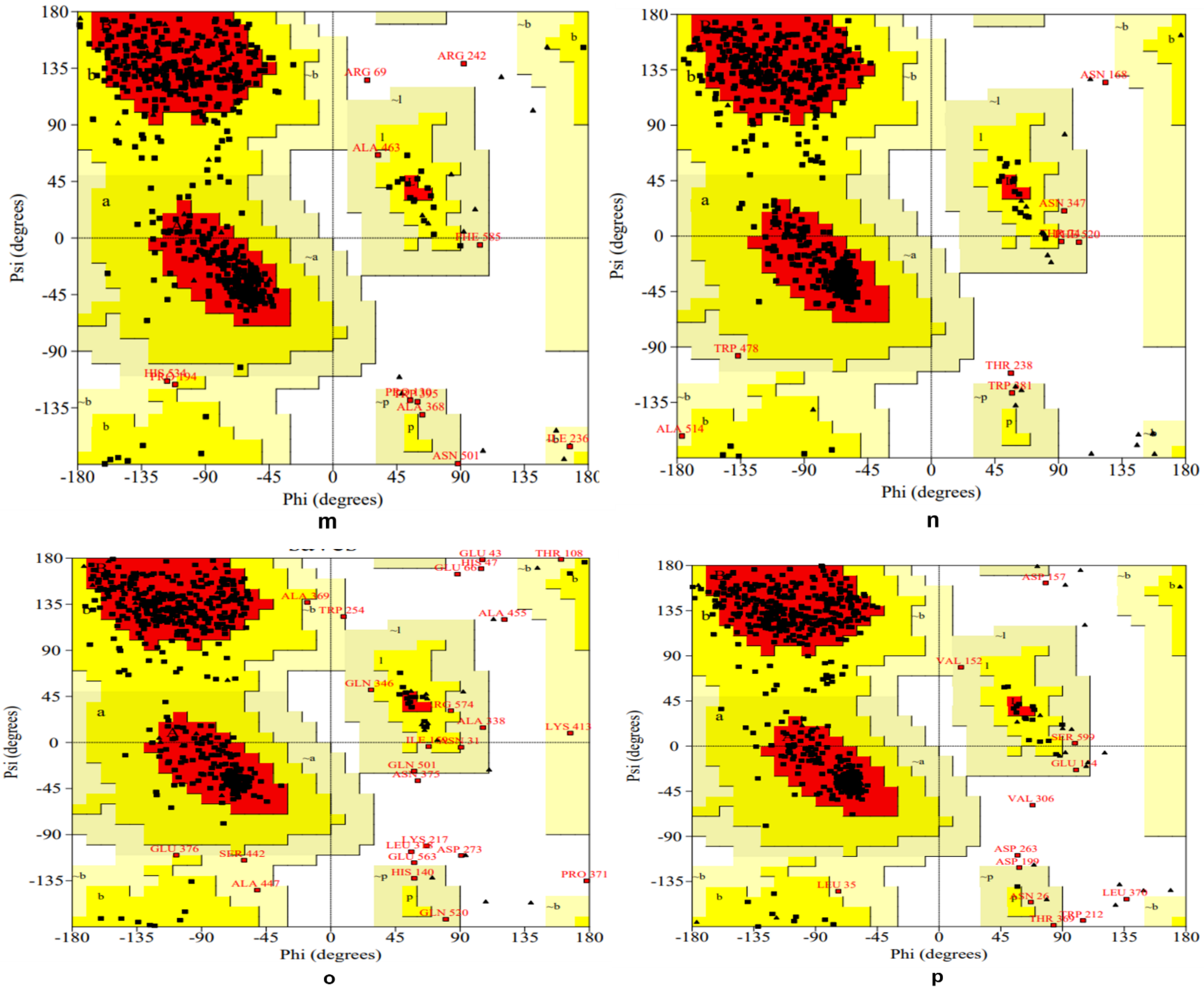
Psi (degrees)
Phi (degrees)
m
n
o
p
ARG 69
ARG 242
ALA 463
PHE 585
HIS 534
PRO 194
ALA 368
ASN 501
ILE 236
ASN 168
ASN 347
TRP 478
THR 238
ALA 514
GLU 43
HIS 47
GLU 66
THR 108
ALA 369
TRP 254
ALA 455
GLN 346
ALA 338
LYS 413
GLN 501
ASN 375
LYS 217
ASP 273
GLU 563
HIS 140
GLU 376
SER 442
ALA 447
PRO 371
GLN 520
ASP 157
VAL 152
SER 599
VAL 306
ASP 263
ASP 199
LEU 35
LEU 370
TRP 212

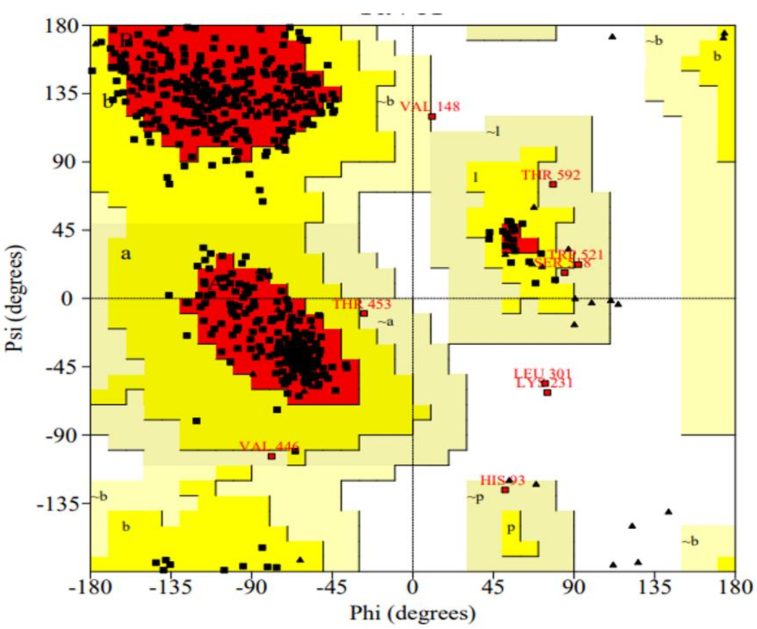
Psi (degrees)
Phi (degrees)
VAL 148
THR 592
TRP 521
THR 453
LEU 301
LYS 231
VAL 446
HIS 93

q

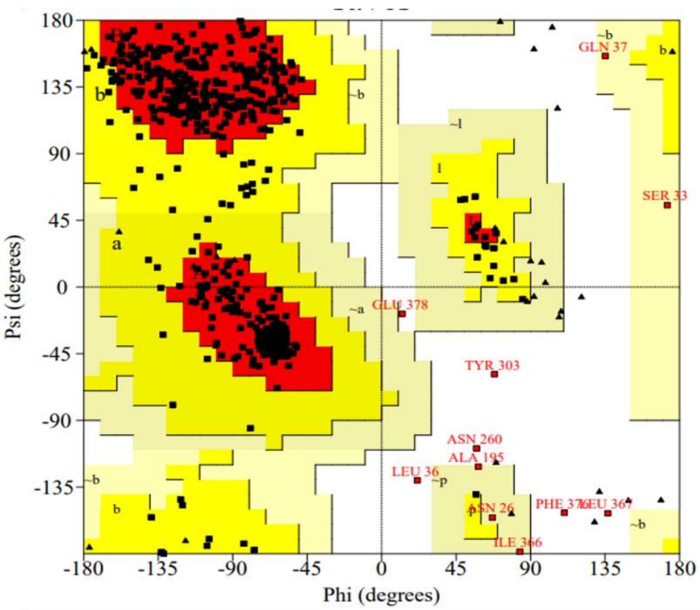
Psi (degrees)
Phi (degrees)
GLN 37
SER 33
GLU 378
TYR 303
ASN 260
ALA 195
LEU 36
ASN 26
LEU 367
ILE 366

r

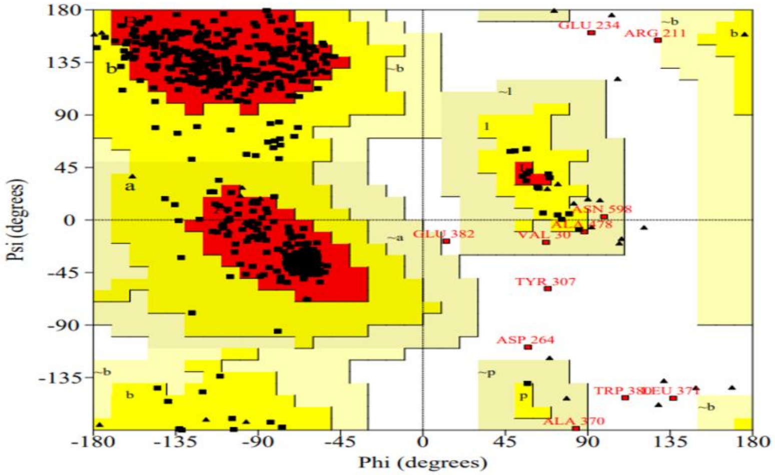
Psi (degrees)
Phi (degrees)
GLU 234
ARG 211
ASN 598
ALA 478
GLU 382
VAL 30
TYR 307
ASP 264
ALA 370

s

Supplementary Data Figure S2: Ramachandran plots predicting quality of GUS protein structures of bacteria discussed in present study
